# Identifying direct temporal relations between time and events from clinical notes

**DOI:** 10.1186/s12911-018-0627-5

**Published:** 2018-07-23

**Authors:** Hee-Jin Lee, Yaoyun Zhang, Min Jiang, Jun Xu, Cui Tao, Hua Xu

**Affiliations:** 1 School of Biomedical Informatics, The University of Texas Health Science Center at Houston, Houston, TX USA; 2 Pieces Technologies, Dallas, TX USA

**Keywords:** Temporal relation identification, Direct temporal relation, Information extraction, TLINK, Syntactic structure

## Abstract

**Background:**

Most of the current work on clinical temporal relation identification follows the convention developed in the general domain, aiming to identify a comprehensive set of temporal relations from a document including both explicit and implicit relations. While such a comprehensive set can represent temporal information in a document in a complete manner, some of the temporal relations in the comprehensive set may not be essential depending on the clinical application of interest. Moreover, as the types of evidence that should be used to identify explicit and implicit relations are different, current clinical temporal relation identification systems that target both explicit and implicit relations still show low performances for practical use.

**Methods:**

In this paper, we propose to focus on a sub-task of conventional temporal relation identification task in order to provide insight into building practical temporal relation identification modules for clinical text. We focus on identification of *direct* temporal relations, a subset of temporal relations that is chosen to minimize the amount of inference required to identify the relations. A corpus on direct temporal relations between time expressions and event mentions is constructed, and an automatic system tailored for direct temporal relations is developed.

**Results:**

It is shown that the direct temporal relations constitute a major category of temporal relations that contain important information needed for clinical applications. The system optimized for direct temporal relations achieves better performance than the state-of-the-art system developed with comprehensive set of both explicit and implicit relations in mind.

**Conclusions:**

We expect direct temporal relations to facilitate the development of practical temporal information extraction tools in clinical domain.

## Background

Clinical narratives are a rich source of information with details about patients’ medical conditions, treatments, and responses that can be utilized for various clinical research projects and applications. Since understanding temporality regarding clinical events conveyed in the narrative text is a crucial prerequisite for the utilization of the narratives, automatic means to identify temporal information from clinical narratives have gained much attention from the community [1–10].

While the task of temporal information identification ranges from identifying time mentions from the text [11, 12] to answering time-related questions [1, 13], this paper focuses on temporal relation identification, which is an essential task in understanding temporality from clinical text. In general, the task of temporal relation identification is formulated as follows: given time expressions and event mentions in clinical narratives, determine what kind of temporal relation does a pair of event mentions and/or time expressions have. Here, a time expression can represent time, date, duration or frequency, such as “7:00 AM”, “Sep. 2. 2016”, “two weeks”, and “daily”, and a clinical event mention can refer to problem, treatment, or test, such as “diabetes”, “metformin”, and “blood glucose test”. For instance, the sentence “The patient underwent the surgery on Tuesday.” contains an event mention “surgery” and a time expression “Tuesday”, and a temporal relation of type “overlap” between the event mention and the time expression.

There are de facto standard corpora on clinical temporal relations that are released through community challenges (i.e., the 2012 Informatics for Integrating Biology and the Bedside (i2b2) challenge [14], the 2013/2014 CLEF/ShARe challenges [4], and the 2015/2016/2017 Clinical TempEval challenges [5–7]). Many systems have been developed based on these corpora [8–10]. Albeit with small differences in the exact annotation guidelines, the corpora are constructed in similar manner. That is, the corpora include implicit temporal relations that are only identifiable through inference, combining multiple pieces of information, as well as temporal relations that are explicitly stated in the text. For instance, temporal relations between mentions across multiple sentences are included. Moreover, since the number of potential temporal relations for a given text is too large (i.e., up to N (N − 1)/2 temporal relations for a document with N mentions [3]), rather than annotating every temporal relation by hand, the notion of transitivity among the temporal relations is introduced. The transitive closure^1^ Of a given set of manually annotated temporal relations is calculated and used as the full set of temporal relations identifiable from a text.

**Fig. 1:**
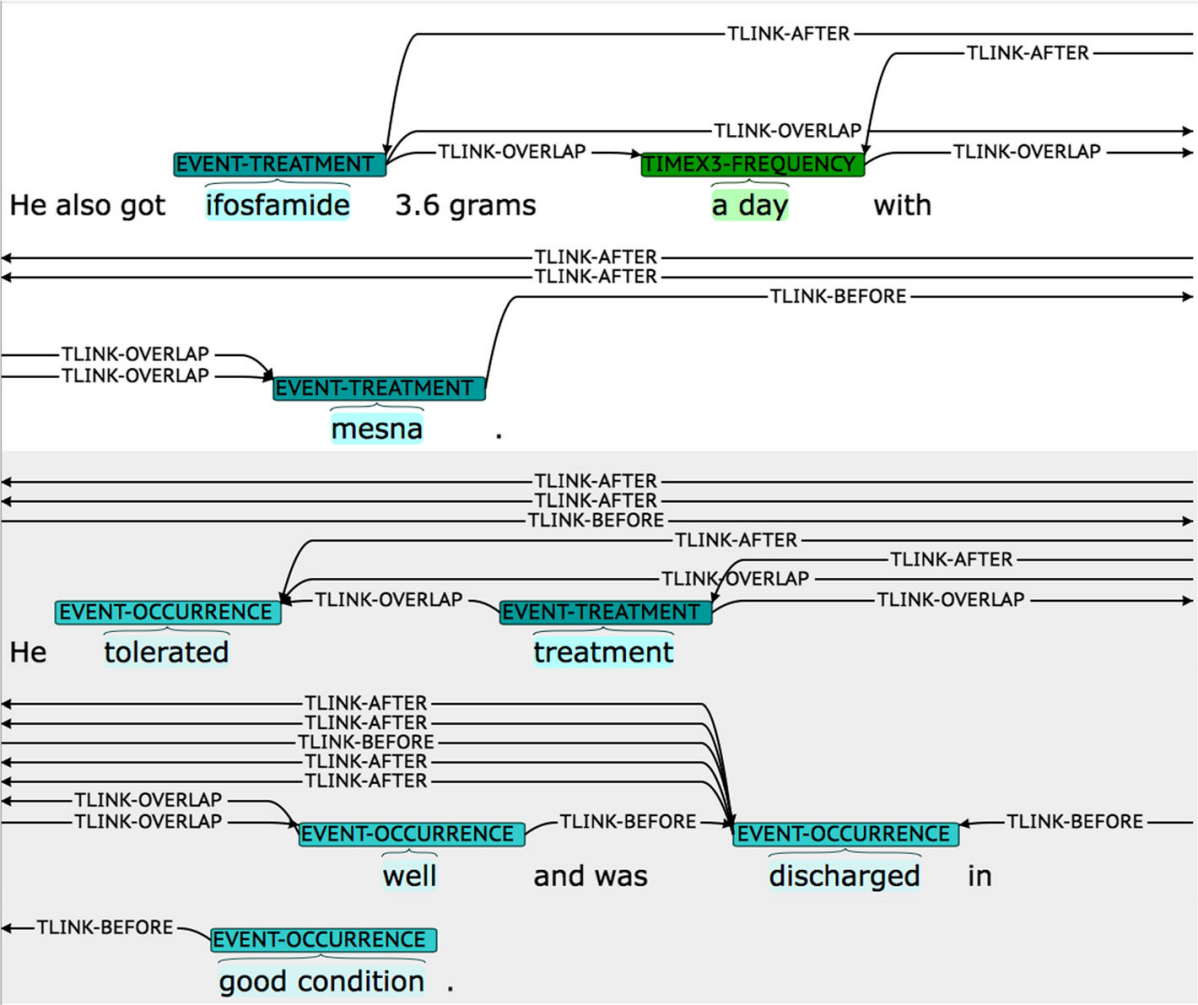
Visualization of a small partial temporal relation network excerpted from the i2b2 2012 corpora [14]. Two sentences contain 8 temporal mentions and 26 temporal relations. For clarity, when there are more than one relations for a pair of temporal mentions, only one of the relations is shown

While such a comprehensive way of representing temporal relations provides a complete view of temporal relations for a given document, the resulting set of temporal relations often comprises an overly complex network (c.f., Fig. 1). Such a complex network is difficult to comprehend for humans, and some part of the network may be non-essential depending on the clinical application of interest. For instance, if one wants to build a timeline of clinical events for a patient, only the relations between a time expression and an event mention would become essential, and identifying other relations between two events or between two time expressions would become less relevant.

Moreover, evidence that can be used to identify explicit or implicit temporal relations is different. While explicit relations can be identified based on textual clues, implicit relations can be identified based on inference combining multiple pieces of temporal information. Thus, mixing the two types of relations makes automatic systems unable to fully utilize the different types of clues, making it difficult to develop optimized NLP methods. Although much work has been done for temporal relation identification from clinical text, the state-of-the-art performance is still not adequate for wide adoption in practical applications; the best systems’ performances for recent challenges have F1-scores of 57.3 [9] and 69.43 [10], respectively. Therefore, it is important to separate the tasks of explicit relation and implicit relation extraction and investigate methods for each type of relations in depth.

In our previous work [15], we proposed to focus on a sub-problem of the standard temporal relation identification task. Instead of targeting all temporal relations identifiable from a text, we focused on a subset of relations between temporal expressions and events. Our subset was chosen to minimize the amount of inference required to identify the relations, so that an automatic method fully utilizing textual evidence can be developed to achieve better performance while maintaining as much of information as possible that is useful in clinical applications. We focused on “direct” temporal relations, which are intra-sentential relations with limited syntactic distance between a temporal expression and an event mention. A small corpus was constructed to perform initial analysis.

In this paper, we extend our previous work to enlarge the corpus of direct temporal relations between time expressions and events by leveraging the 2012 i2b2 challenge corpus. In addition, a Support Vector Machine (SVM)-based system optimized for direct temporal relations is developed. Our analysis shows that the direct temporal relations constitute a major category of temporal relations, and the system tailored to direct temporal relations shows much better performance on direct temporal relations than a state-of-the-art system developed for the complete temporal relation identification task.

## Related work

The task of temporal relation identification from clinical narratives has been tackled with various approaches, including machine-learning frameworks such as SVM [8–10], Markov Logic Network (MLN) [16], and structured learning [17]. In many systems, the entire set of temporal relations is often decomposed into several groups based on their characteristics. For instance, the Vanderbilt system [10] divides the temporal relations into six groups (i.e., event-admission time relations, event-discharge time relations, intra-sentential event-event relations, intra-sentential event-time relations, inter-sentential relations across consecutive sentences, and inter-sentential relations with co-references), and trains a separate SVM classifier for each group. Similar approach is adopted by other systems that use SVM [8, 9]. These systems differentiate intra-sentential relations from inter-sentential relations, but do not differentiate implicit relation within a sentence from explicitly stated relations.

Some focus on identifying implicit relations. Xu et al. [16] train 10 separate SVM classifiers to identify both explicit and implicit relations, and then apply MLN to further infer implicit relations based on the results produced by the SVM classifiers. Leeuwenberg and Moens [17] use structured perceptron model that jointly learns the relations between events and the document-creation time and the relations between events and time expressions in the text. The model training and prediction is done at a document level using global features that can exploit local evidences. While these systems report increased performance with enhanced identification of implicit relations, the systems do not include any specialized method for explicit relations.

The rest of this paper is organized as follows: in the METHODS Section, we first introduce direct temporal relation and describe the procedure to construct a corpus of direct temporal relations (Section Direct Temporal Relations). And then, we introduce an automatic relation identification system tailored to the direct relations (Section Automatic Identification System). After that, we detail the experiments done in this paper (Section Experimental Setup). The results of the experiments are reported in Section RESULTS, followed by Section DISCUSSION and Section CONCLUSION.

## Methods

### Direct temporal relations

**Fig. 2:**
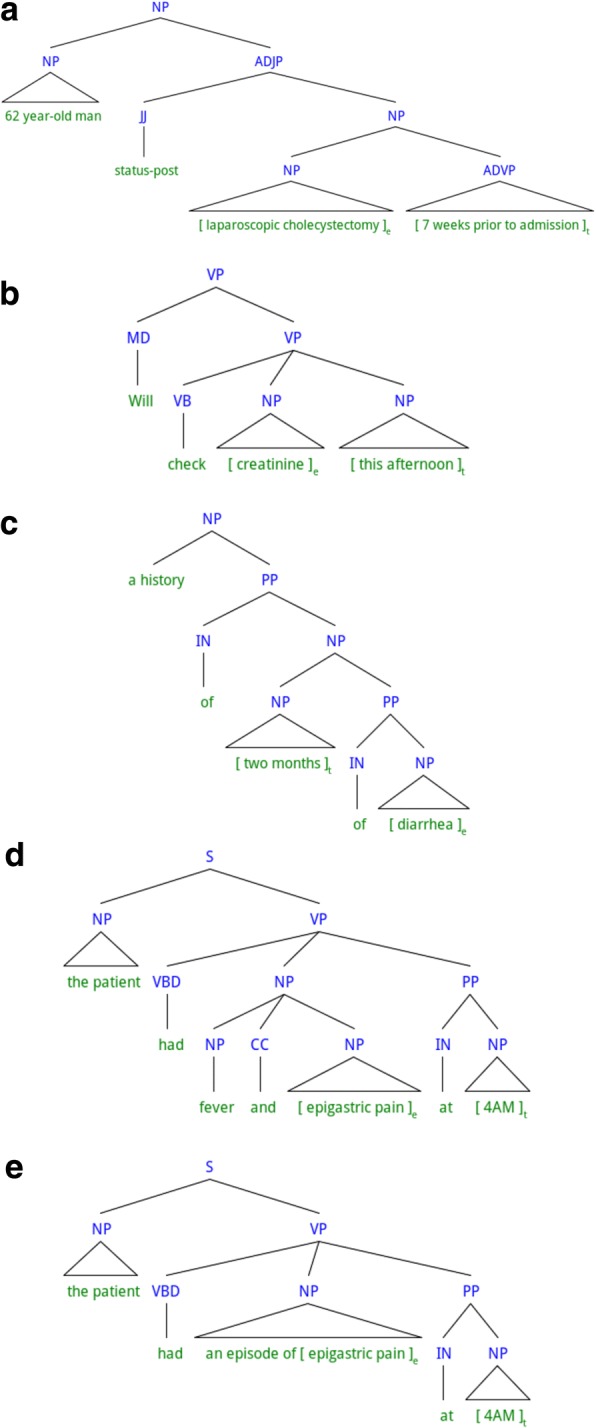
Examples of direct temporal relations. Five examples with labels, **a**, **b**, **c**, **d**, **e**, are illustrated

**Fig. 3:**
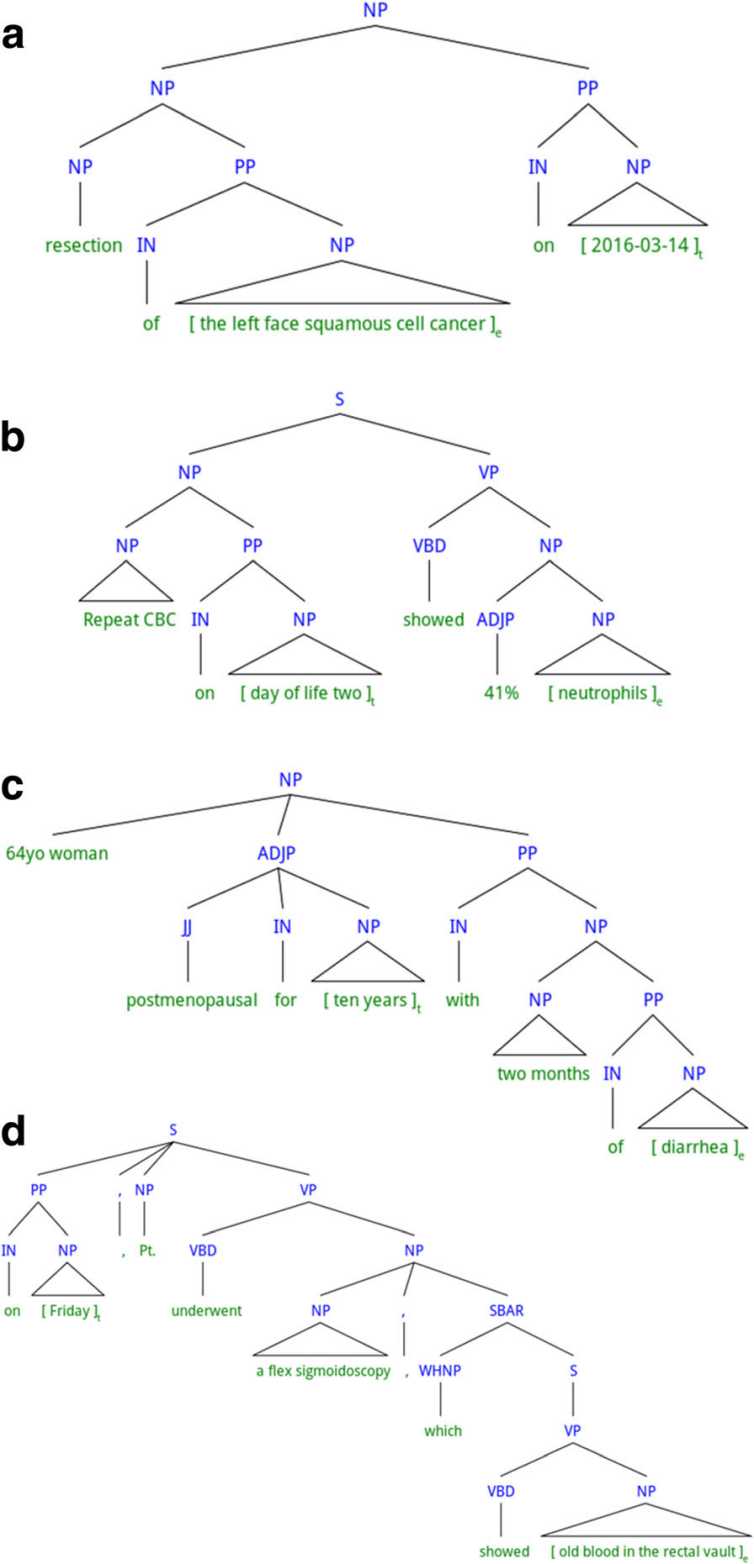
Examples of non-direct temporal relations. Four examples with labels, **a**, **b**, **c**, **d**, are illustrated

In this section, we define direct temporal relation in Section *Definition*, and detail the procedure to construct the direct temporal relation corpus in Section *Corpus construction*. Examples of direct and non-direct temporal relations are shown in Fig. 2 and Fig. 3, respectively. In the examples, a constituency parse tree of the target sentence is shown as our definition of direct temporal relation is based on constituency-based syntax, and the target time expression and the target event mention are marked with square brackets and subscripts ‘t’ and ‘e’, respectively. Note that non-direct temporal relations refer to the temporal relations that are not direct.

#### Definition

We define a direct temporal relation as either 1) a temporal relation whose time expression modifies the event mention (or vice versa), or 2) a temporal relation whose time expression and event mention are arguments or adjuncts of the same predicate. Here, a temporal relation is an ordered relation between a time expression and an event mention, which can have one of the following three types: “before”, “after”, or “overlap”. The types of the temporal relations follow the types used in the 2012 i2b2 challenge. While conventional temporal relation work covers temporal relations between two event mentions as well, our direct temporal relations focus on temporal relations between a time expression and an event mention.

Modification of an event mention by a time expression refers to a syntactic construction that the event mention is accompanied (or modified) by the time expression to form a bigger grammatical element. For instance, for the example in Fig. 2(a), the target time expression “7 weeks prior to admission” forms an adverbial phrase (ADVP), and the target event mention “laparoscopic cholecystectomy” forms a noun phrase (NP). The ADVP containing the time expression is modifying the NP containing the event mention, forming a bigger NP “laparoscopic cholecystectomy 7 weeks prior to admission”. Thus, by the first rule of the definition of direct temporal relations, the time expression “7 weeks prior to admission” and the event mention “laparoscopic cholecystectomy” form a direct temporal relation.

Usually, a verb or a noun is considered as a predicate in linguistics. A predicate needs one or more arguments acting in different syntactic/semantic roles to complete its meaning. Adjuncts are another type of grammatical elements used to modify and complete the meaning of predicates. Different from arguments, adjuncts can be removed from a sentence without making it grammatically wrong. Taking Fig. 2(b) as an example, the NP formed by the mention of a lab test event “creatinine” serves as an object of the verb “check”. Meanwhile, the NP formed by the time expression “this afternoon” modifies the same verb “check” as an adjunct, providing additional information on when the event of “check creatinine” will happen. Thus, according to the second rule of the definition of direct temporal relations, the time expression and the event mention in Fig. 2(b) form a direct temporal relation.

Note that the time expression and the event mention should be the head of the phrase that modifies the other phrase (or being modified by the other phrase), or the phrase serving as an argument or an adjunct of the predicate. Head of a phrase is a sub-phrase that determines the syntactic type of the phrase. Every event mention and time expression shown in Fig. 2(a) and (b) constitutes a NP by itself, thus all of them are heads of the respective NPs. By contrast, in the example shown in Fig. 3(a), the time expression “2016–03-14” is part of a prepositional phrase (PP) “on 2016–03-13”, and the event mention “the left face squamous cell cancer” is part of a NP “resection of the left face squamous cell cancer”. The PP modifies the NP, but the event mention is not the head of the NP (“resection” is the head), thus, the time expression and the event mention in this example cannot form a direct temporal relation. Similarly, in Fig. 3(b), the time expression “day of life two” is contained in a NP “repeat CBC on day of life two”, and the even mention “neutrophils” is contained in a NP “41% neutrophils”. The two NPs are both arguments of the same verb “showed”. However, the time expression is not the head of the NP (“repeat CBC” is the head), thus, the time expression and the event mention cannot form a direct temporal relation. Examples of non-direct temporal relations with more complex sentence structures are shown in Fig. 3(c) and (d).

However, there are cases where even when the time expression or the event mention is not the head of the respective phrase, it is rather straightforward to identify the temporal relation (i.e., not requiring inference that combines multiple pieces of information). Since our initial goal was to identify a subset of temporal relations that requires less number of inferences to identify but still contains as much important information as possible, we make exceptions to the above “head-of-the-phrase” rule.^2^ There are three cases in which the pairs are allowed to form direct temporal relations even when the time expressions and the event mentions are not the heads of the phrases:

- Case 1: The time expression or the event mention is contained inside a PP, which can be decomposed into a preposition (which is the head of the PP) and a NP. If the time expression or the event mention is the head of the NP, then the time expression or the event mention is allowed to form a direct temporal relation.
- Case 2: The time expression or the event mention is contained inside a coordinated NP, which contains multiple smaller NPs (coordinates) and conjunction words (e.g., “and”, “or”, “,”). If the time expression or the event mention is the head of one of the coordinates (smaller NPs), then the time expression or the event mention is allowed to form a direct temporal relation.
- Case 3: The time expression or the event mention is the head of a phrase P, and P is contained in a bigger phrase which is one of the type-preserving phrases (c.f. Table 1).

**Table 1:** Examples of type-preserving phrases

Template^a^	Examples^b^
the first course of [Event-Treatment]	the first course of *Velban*
episodes of [Event-Problem]	episodes of *gastric pain*
the time of [Time-Date]	the time of *3/21*
period of [Time-Duration]	period of *14 days*

^a^In the templates, squared parentheses mark the placeholder for a time expression or an event mention

^b^In the examples, italicized letters mark a time expression or an event mention

The example in Fig. 2(c) shows a pair with case 1. The time expression “two months” itself is a NP, which is modified by a PP “of diarrhea”. Although the event mention “diarrhea” is not the head of the PP (the preposition “of” is the head), by exception case 1, this pair can form a direct temporal relation. An example pertinent to case 2 is shown in Fig. 2(d). The verb “had” has the NP “fever and epigastric pain” as an argument and the PP “at 4 AM” as an adjunct. Although the event mention “epigastric pain” is not the head of the NP “fever and epigastric pain”, it can form a direct temporal relation, since the NP “fever and epigastric pain” is coordinated and the event mention “epigastric pain” is one of the coordinates.

A “type-preserving phrase”, as mentioned in case 3, is a phrase whose semantic type can be regarded as time (i.e., time, date, duration, or frequency) or clinical event (i.e., problem, treatment, or test), even though its head is not a time expression or an event mention. For instance, for the NP “an episode of diarrhea”, “an episode” is the head of the phrase, but the phrase can be regarded as conveying the meaning of a problem type clinical event, “diarrhea”. Thus, the phrase can be a type-preserving phrase. In order to avoid confusion during annotation, a pre-compiled list of type-preserving phrases is added to the annotation guideline. Table 1 shows some of the type-preserving phrases. During annotation, type-preserving phrases are manually identified given types of time expressions and event mentions (i.e., “time”, “date”, “duration”, or “frequency” for time expressions and “problem”, “test”, or “treatment” for event mentions). In Fig. 2(e), the problem type event mention, “epigastric pain”, is contained inside a type-preserving phrase “an episode of epigastric pain”, which serves as an argument of the verb “had”. Although the even mention is not the head of this phrase, by exception case 3, the pair shown in Fig. 2(e) can form a direct temporal relation.

#### Corpus construction

**Fig. 4:**
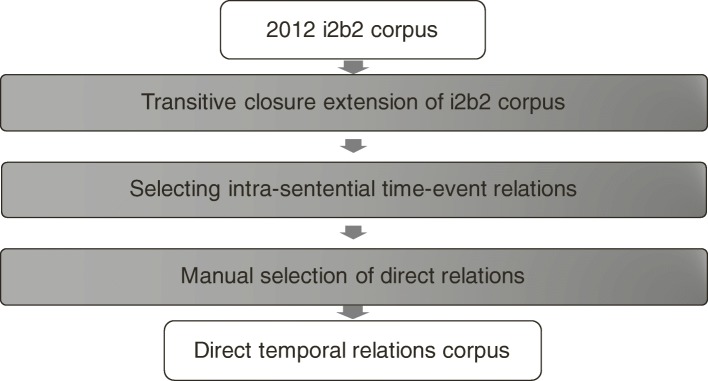
The process of direct temporal relations corpus construction

A corpus of direct temporal relations is constructed by leveraging the 2012 i2b2 corpus. The corpus construction process is shown in Fig. 4. First, the transitive closure of the 2012 i2b2 corpus is calculated to produce the set of all inferable temporal relations from the 2012 i2b2 corpus. The rules for transitive closure calculation are shown in Table 2. Second, only the intra-sentential temporal relations between a time expression and an event mention is selected. Finally, each intra-sentential time-event temporal relation is reviewed by domain experts to be decided whether it is a direct temporal relation or not. The experts followed the definitions in Section *Definition* and the annotation guidelines^3^ that were developed through the pilot annotation phase with small number of documents. The whole 2012 i2b2 corpus (310 discharge summaries, including both training and test set) is reviewed by an expert. Randomly selected 16 discharge summaries containing 383 pairs of time expressions and event mentions are reviewed by another expert, to measure the inter-annotator agreement rate. After manual review, only the direct temporal relations are collected. As a result, a new corpus of direct temporal relations is constructed. Note that the direct temporal relations preserve the temporal relation type (i.e., “after”, “before”, or “overlap”) from the original 2012 i2b2 corpus.

**Table 2:** Rules for transitive closure calculation^a^

- If A overlap B, then B overlap A - If A before B, then B after A - If A after B, then B before A - If A overlap B, and B overlap C, then A overlap C - If A before B, and B before C, then A before C - If A before B, and B overlap C, then A before C - If A overlap B, and B before C, then A before C	

^a^It is possible that the rules may produce false positive or false negative temporal relations. For instance, A and C may not overlap even when A overlap B and B overlap C. The false positive relations are removed and missing relations are added during the manual annotation process by the domain experts

### Automatic direct temporal relation identification system

**Fig. 5:**
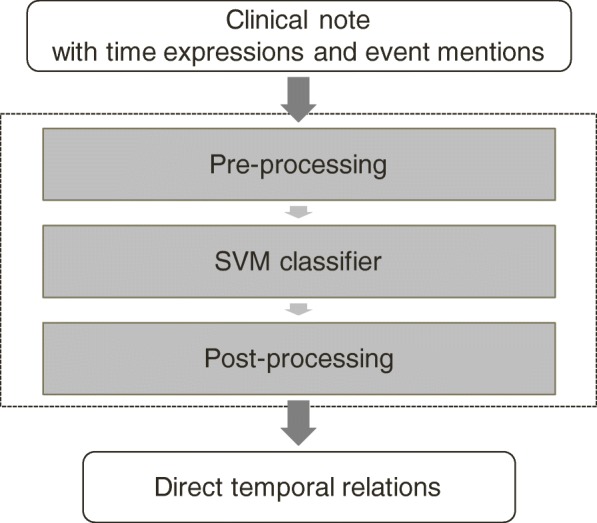
Structure of the direct temporal relation identification system

In this section, we introduce a SVM-based system tailored to direct temporal relations. The system takes as input a document with annotations of time expressions and event mentions, and outputs direct temporal relations found in the document. The system is composed of three parts: a pre-processing module, a SVM classifier, and a post-processing module. Fig. 5 shows the structure of the system. We chose to use SVM as the classification algorithm since it has shown good performances in many temporal relation identification systems [8–10].

The pre-processing module includes tokenization and section identification by the CLAMP tool [18], POS (Part Of Speech) tagging by OpenNLP [19], dependency parsing by ClearTK [20], and semantic role labeling by SENNA [21].

The SVM classifier determines if a candidate intra-sentential pair of a time expression and an event mention forms a direct temporal relation or not, and classifies the pair into “overlap”, “before”, or “after” (when the pair forms a direct temporal relation), or “no-relation” (when the pair does not form a direct temporal relation). Since direct temporal relations are defined based on syntactic structure and predicate-argument structure, features derived from parse trees and semantic role labels are included in addition to the features that have shown to be effective for clinical temporal relation identification [9, 10]. The features are listed below:

1. Time expression type: one of “date”, “time”, “duration”, and “frequency”, as given in the original i2b2 corpus
2. Event mention attributes: “type”, “polarity”, “modality” of the event mention, as given in the original i2b2 corpus
3. Tokens of the mentions and the context: bag of tokens of the time expression and the event mention, preceding and following three tokens of the mentions, bag of tokens in-between the mentions, number of tokens in-between the mentions
4. POS tags: POS tags of the tokens of the mentions
5. Punctuations: number of punctuations in-between the mentions
6. Verb tense: tense and position of the verbs in the sentence
7. Section type: type of the section in which the target mentions are found, e.g., “History of present illness”, “Brief hospital course”
8. Sentence type: if the sentence starts with an enumeration mark such as “2.”, and “a)”, or if the sentence ends with a colon
9. Dependency features: common ancestor of the time expression and the event mention on the dependency parse tree of the sentence, whether a noun exists or not in the dependency path from the event mention to the time expression
10. Semantic roles: predicates of the time expression and the event mention, whether the time expression and the event mentions are arguments/adjuncts of the same predicate or not

Since the type distribution of the direct temporal relations is imbalanced (c.f. Table 3), cost-sensitive learning is applied by assigning weights to each type that are inversely proportional to the type frequency. LibSVM [22] implementation of SVM is used.

**Table 3:** Type distribution of direct temporal relations

Temporal relation type	Training set	Test set	Overall
Before	387 (17%)	355 (20%)	742 (18%)
After	345 (15%)	299 (16%)	644 (16%)
Overlap	1518 (68%)	1173 (64%)	2690 (66%)
total	2249 (100%)	1827 (100%)	4076 (100%)

Lastly, the post-processing step consists of deterministic rules to fix common errors observed during the development period. For instance, a direct temporal relation is regarded as a false positive and removed from the final output when the relation is between a problem type event mention and a frequency type time expression, and the time expression represents frequency of medication (e.g., “bid”, “prn”, or “q2h”). Similarly, a direct temporal relation is regarded as a false positive if there’s a word “where” in-between the time expression and the event mention of the direct temporal relation.

### Experimental setup

In order to examine the extent of coverage of direct temporal relations in the 2012 i2b2, we compare the direct temporal relation corpus to the 2012 i2b2 corpus. Similar to the evaluation method in the i2b2 challenge [14], the set of direct relations is first extended to its transitive closure, and then the extended set is compared to the i2b2 annotations.

The performance of the SVM-based system that is developed specifically for the direct temporal relations is reported. The performance is also compared to other baseline systems. We employ three baseline systems: 1) the Vanderbilt system [10], which is the best-performing system in the 2012 i2b2 challenge, 2) a syntactic graph kernel based system [23], and 3) a Conditional Random Fields (CRF)-based system [24]. The Vanderbilt system is the state-of-the-art system that is developed for the complete set of temporal relations (i.e., including both explicit and implicit relations). We re-implement the system and re-train it with direct temporal relations corpus. Such an evaluation provides insight on how effective the Vanderbilt systems features (i.e., features for machine learning and other gazetteers/rules) are for identification of direct temporal relations. In addition, we also evaluate the results produced by the Vanderbilt system for the 2012 i2b2 challenge (i.e., the submission by the Vanderbilt system to the TLINK-only track) against direct temporal relations. This second evaluation of the Vanderbilt system provides a view on how accurately the direct temporal relations are handled among the complete set of temporal relations in the standard task of temporal relation identification. Since the submission contains non-direct temporal relations as well as direct temporal relations, a procedure similar to that of temporal relations corpus construction is adopted: 1) the transitive closure of the Vanderbilt systems output is calculated, 2) only the relations between direct pairs of a time expression and an event mention are kept (i.e., temporal relations between pairs that do not conform to the rules for direct temporal relations are excluded), and 3) the resulting set of temporal relations is compared to the direct temporal relations corpus for evaluation. Syntactic graph kernel based methods [23, 25] are shown to achieve high performance for relation extraction tasks when the target relations are highly dependent on syntactic structure. Thus, the method is expected to perform well for direct temporal relations. CRF-based methods [24, 26] are shown to achieve good performance when the surface distance between the two entities of relation is short, thus selected as another strong baseline.

## Results

In this section, we report the results of our experiments and discuss the results.

### Statistics on the direct temporal relation corpus

The direct temporal relation corpus contains 310 discharge summaries. The discharge summaries are split into a training set of 190 documents and a test set of 120 documents following data split in the original 2012 i2b2 corpus (i.e., the documents in the training and test sets of direct temporal relation corpus are the same as the documents in the training and test sets of the original 2012 i2b2 corpus, respectively). Table 3 shows the type distribution of direct temporal relations in the corpus. The type distribution is similar to the type distribution of the 2012 i2b2 corpus (71.1% of the temporal relations have type “overlap”, and the rest “before” or “after”).

The inter-annotator agreement rate between the two experts is shown to be 80.10 (Cohens kappa [27]), which represents a good agreement.

### Comparison to the standard temporal relation identification task

The direct temporal relations in our corpus constitutes 88.75% of all intra-sentential temporal relations between a time expression and an event mention in the 2012 i2b2 corpus. The direct relations also constitute 73.37% of all the temporal relations between a time expression and an event mention in the 2012 i2b2 corpus. This indicates that the direct temporal relations constitute a major category of temporal relations.

The performance of the Vanderbilt system (using submission to the 2012 i2b2 challenge) on direct temporal relations is precision 43.53, recall 76.99, and F1-score 55.61, indicating the need for the development of methods specialized for direct temporal relations.

### Automatic identification performance

The performance of the SVM-based direct temporal relation identification system is shown in Table 4, along with the performances of other systems. The F1-score of the SVM-based system is shown to be 63.77, the best among all the systems. Specifically, the performance is much higher than the F1-score of the original Vanderbilt system (55.61) or the re-implemented Vanderbilt system (55.66). This shows that a system specialized for the direct temporal relations can achieve much better performance than the state-of-the-art system developed for the standard temporal relation identification task. The original Vanderbilt system shows best recall, probably due to the fact that the system is developed for the entire set of temporal relations. Syntactic graph kernel shows second-best F_1_-score and best precision, showing that syntactic information is indeed important in identifying direct temporal relations. On the other hand, the CRF-based system performs poorer than the other systems. We conjecture that such a poor performance is due to the fact that the CRF-based system does not utilize any syntactic-level information.

**Table 4:** Performances of automatic direct temporal relation identification systems

System	P	R	F_1_
SVM-based system	63.93	63.62	**63.77**
Original Vanderbilt system (submission to 2012 i2b2 challenge)	43.53	**76.99**	55.61
Re-trained Vanderbilt system (re-impremented, re-trained on direct temporal relation corpus)	64.16	49.15	55.66
Syntactic graph kernel based system	**64.46**	54.27	58.92
CRF-based system	48.51	39.52	43.56

Best scores for precision, recall and F_1_-score are marked bold

Table 5 shows example outputs of the SVM-based system. The table shows both correctly identified direct temporal relations and erroneous outputs.

**Table 5:** Example outputs of svm-based system

Sentence	Predicted	Gold standard
Subsequently [his creatinine]_e_ rose for [three days]_t_ and then stabilized at 10.	overlap	overlap
[Cardiac catheterization]_e_ was performed without complication from the right neck and right groin on [the day]_t_ of admission.	overlap	overlap
3. On [the morning of 12–01]_t_, the patient had some transient episodes of hypotension with [SBP s]_e_ in the 70 s.	overlap	N/A
Initially on [Vanc]_e_ and Cipro on [Friday]_t_ but then seen by ID who recommended no abx but a bone biopsy, blood cx.	N/A	overlap
Anti-coagulation was started with Warfarin 5 mg with a goal of 2–3 and a plan for [cardioversion]_e_ in [6 weeks]_t_.	overlap	after
[POD# 15/6]_t_, she resumed [TF]_e_ ‘s and TPN was tapered again.	overlap	after

In the “sentences”, the target time expression and the target event mention are marked with square brackets and subscripts ‘t’ and ‘e’, respectively. “Predicted” is the type of direct temporal relation predicted by the SVM-based system. “Gold standard” is the gold standard type of direct temporal relation. When the temporal relation is non-direct, it is represented as “N/A”

## Discussion

In this paper, we focused on direct temporal relations, instead of targeting the entire set of inferable temporal relations from a document as done in the standard temporal relation identification tasks. We defined direct temporal relation based on the syntactic structures and shallow semantic structures of the sentences, and our goal was to limit the amount of inference required to identify the temporal relations. A corpus of direct temporal relations was constructed by leveraging an existing corpus. An automatic system optimized for the direct temporal relations was developed using the new corpus. We showed that the direct temporal relations constitute a major category of temporal relations, and the automatic identification system optimized for direct temporal relations achieved much better performance than the state-of-the-art system that is developed for the complete temporal relation identification task.

The performance of the original Vanderbilt system (using the submission to the i2b2 challenge) on direct temporal relations is reported to be F_1_-score 55.61 (Table 4). Interestingly, the performance of the system on the entire set of temporal relations (i.e., the official TLINK-only track record of the system) is shown to be F_1_-score 69.32 [10], which is much higher than the system’s performance on direct temporal relations. In fact, the entire set of temporal relations provided by the 2012 i2b2 challenge corpus contains relations between clinical event mentions and section times (i.e., admission time and discharge time as the 2012 i2b2 corpus is composed of discharge summaries). Such relations to section times constitute almost half of the entire temporal relations (45.87%), and are shown to be much easier to identify than other types of temporal relations [14]. This demonstrates the complexity of the complete temporal relation extraction task and the need to separate the entire task into different sub-tasks and develop optimized methods for each of the sub-tasks.

The SVM-based direct temporal relation identification system shows much better performance than other baseline systems, but the performance is still not ideal for practical use (F_1_-score 63.77). During the development of the system, we identified three major obstacles hindering accurate identification of direct temporal relations. The first is difficulty in identifying the correct syntactic structures of the sentences, particularly, of ambiguous sentences such as the followings:

1. On [post-op day #3]_t_, the patient’s pacing wires were removed, and his [17opressor]_e_ was started.
2. The patient presents with a [four day]_t_ prodrome of dry cough, rhinorrhea, chills, loose bowel movements with diarrhea and no blood, [decreased urine output]_e_, no sick contacts.

For the first sentence, it is ambiguous whether the PP “On post-op day #3” is an adjunct of both of the verbs “removed” and “started”, or of only the first verb “removed”. For the second sentence, it is ambiguous how many of the symptoms listed in this sentence are included in the “four day prodrome” (i.e., is it four day prodrome of dry cough only? Or of dry cough and all other symptoms mentioned in the sentence?). Such ambiguity was in fact a major source of disagreement among the human annotators.

In addition, long sentences with complex syntactic structures often produced errors, probably due to the false parse trees produced by the automatic parser. For example, for the pair of a time expression and an event mention shown in the following sentence, the system missed a direct temporal relation of type “overlap”.

1. On 9–28-92, the patient will return for chemotherapy and she will follow up with her primary doctor, Dr. Jescspald, for [repeat PT]_e_ and Coumadin dosing on [Monday, 9–14-02]_t_.

The second difficulty in accurately identifying direct temporal relations is the required use of common/domain knowledge in assigning appropriate types to direct temporal relations. The direct temporal relations are defined in a syntactically motivated way. Thus, the classification of a temporal relation into a direct or non-direct one can be done based on the constituency parse tree or the predicate-argument structure of the sentence. However, assigning type (i.e., “after”, “before”, “overlap”) to the direct temporal relations still often requires inference based on common and domain knowledge. For instance, consider the three sentences following:

1. On [post-op day#2]_t_, [chest tubes]_e_ were removed.
2. [The next day]_t_, he developed [orthostasis]_e_.
3. [Friday]_t_, the patient underwent [a flex sigmoidoscopy]_e_.

The three sentences above have similar predicate-argument structure to one another; both the time expression and the event mention are arguments/adjuncts of the main verb of the sentence. Thus, all three pairs of time expression and event mention form direct temporal relations. However, the type of the temporal relation differs from one another depending on the meaning of the sentence. For the first sentence, the type of the temporal relation between the treatment event “chest tube” and the time expression “post-op day #2” should be “before”, since one can infer that “chest tubes” was applied before “post day #2”. For the second sentence, the type of the temporal relation should be “after”, since “orthostasis” will remain until being treated starting from “The next day”. Finally, for the third sentence, the type of the temporal relation should be “overlap”, since “sigmoidoscopy” will start and end on “Friday”. In fact, the performance of the SVM-based system on detecting direct temporal relations not considering type assignment is shown to be much higher (precision 78.80, recall 84.33, and F1-score 81.47) than the performance of identifying direct temporal relations considering types. This indicates that type assignment is indeed a major source of error in direct temporal relation identification. We plan to devise methods that can incorporate common/domain knowledge into the direct temporal relation identification process.

Note that the inference here that uses common and domain knowledge is different from the kind of inference that is required to identify non-direct implicit relations such as inter-sentential relations. In order to identify non-direct implicit relations, one needs first to identify multiple direct temporal relations and then perform inference combining the direct relations. In this way, the direct temporal relations can be viewed as the basic building blocks that can be used to infer the complete set of all inferable temporal relations.

The last difficulty in accurately identifying direct temporal relations is the imbalanced nature of the direct temporal relations corpus (c.f., Table 3). Although cost-sensitive learning is applied to counter-balance the imbalance in the dataset, the performance of the minor relation types, “after” and “before”, is much lower than the major type, “overlap” (Table 6). We plan to incorporate synthetic data generation techniques such as SMOTE algorithm [28], in order to improve the performance on minor types.

**Table 6:** Performance of svm-based system for each relation type

Temporal relation type	P	R	F_1_
After	53.44	33.78	31.39
Before	56.87	42.21	48.46
Overlap	66.74	77.66	71.79

Lastly, we discuss on some real example outputs produced by the SVM-based system. See Table 5 of the Automatic identification performance Section for the examples. The first and second examples of Table 5 show direct temporal relations of type “overlap” that are correctly identified by the SVM-based system. Interestingly, all other baseline systems failed to identify the “overlap” type direct temporal relations for these two examples. One possible reason for such difference is that the SVM-based system is the only system that utilizes predicate-argument structure as feature; all other baseline systems do not utilize predicate-argument structure information. Note that in both examples, the event mention and the time expression are arguments or adjuncts of the same predicates (i.e., in the first example, the event mention “his creatinine” and the time expression “three days” are argument and adjunct of the verb predicate “rose”; in the second example, the event mention “Cardiac catheterization” and the time expression “the day” are argument and adjunct of the verb predicate “performed”.).

In the third example of Table 5, the system wrongly classified a non-direct temporal relation between the event mention “SBP s” and the time expression “the morning of 12-10” as a direct temporal relation of type “overlap”. We conjecture that such an error is produced by the failure in correctly identifying the syntactic tree structure of the sentence (i.e., attachment of the PP “with SBPs in the 70s” to the verb “had” instead of the noun “hypotension”). In the fourth example, the system failed to identify an “overlap” type direct temporal relation between the event mention “Vanc” and the time expression “Friday”. Again, this error seems to be due to the failure in correctly identifying syntactic structure of the sentence (i.e., attachment of the PP “on Friday” to the NP “Cipro” instead of the NP “Vanc and Cipro”).

For the last two examples, the system incorrectly identified direct temporal relations of type “after” as direct temporal relations of type “overlap”. For the fifth example, while the preposition “in” usually signals temporal relation of type “overlap”, in this context it conveys the meaning that “cardioversion” would be performed after a time period of “6 weeks”. The system failed to identify such information. For the last example, the verb “resume” conveys the information that “TF” started on “POD# 15/6” and continued for some time after “POD# 15/6”. However, the system failed to identify this information. In fact, all other baseline systems also failed to identify correct direct temporal relations for these two examples.

As future work, we plan to investigate different ways to improve the performance of automatic direct temporal relation identification. We plan to investigate how different features contribute to the performance, and to test diverse types of machine learning algorithms including deep learning. We are also considering using a rule-based system instead of machine-learning based systems as our definition of direct temporal relations describes a deterministic process of direct temporal relation identification based on syntactic structures of the sentences.

## Conclusion

In this paper, we proposed to focus on direct temporal relations instead of targeting all inferable relations from a document. A corpus of direct temporal relations is constructed, and an automatic system that is tailored to the direct temporal relations is developed. It is shown that the direct temporal relations constitute a major category of temporal relations, and that a system optimized for direct temporal relations can achieve much better performance than a state-of-the-art system targeting all inferable temporal relations. We expect methods for direct temporal relations to advance wide adoption of automatic temporal information extraction tools in practical medical applications.

## Footnotes

1 Transitive closure of a set of temporal relations is the minimal set of minimal transitive relations that contains the original set of temporal relations.

2 Based on our annotation, about 26% of direct temporal relations strictly follow the “head-of-the-phrase” rule, and the rest of direct temporal relations are identified based on the exception cases.

3 The annotation guideline is available at https://sbmi.uth.edu/ccb/resources/temporal.htm.
